# A Mendelian randomization-based approach to explore the relationship between leukocyte counts and breast cancer risk in European ethnic groups

**DOI:** 10.1038/s41598-023-44397-9

**Published:** 2023-10-09

**Authors:** Zhitao Zhang, Lei Li, Jianbin Wu

**Affiliations:** 1 Department of Breast, Fujian Maternity and Child Health Hospital, Fuzhou, China; 2 Department of Pathology, University of Otago, Dunedin, 9016 New Zealand

**Keywords:** Cancer, Computational biology and bioinformatics, Medical research, Oncology, Risk factors

## Abstract

Exploring the potential association between peripheral blood leukocyte counts and breast cancer risk by Mendelian randomization (MR) analysis methods. Genetic data related to peripheral blood sorting counts of leukocytes were collected from a genome-wide association study by Blood Cell Consortium (BCX). Single nucleotide polymorphic loci predicting peripheral blood sorting counts of these leukocytes were selected as instrumental variables according to the correlation assumption, independence assumption and exclusivity assumption of MR. The data on breast cancer and its subtypes were obtained from Breast Cancer Association Consortium (BCAC) and FinnGen Consortium. In this study, the Inverse-Variance Weighted (IVW), Weighted Median, MR-Egger, Maximum Likelihood (ML), MR-PRESSO and Constrained Maximum Likelihood and Model Averaging (cML-MA) methods of random effects models were used for MR analysis. Cochran’s Q analysis, and MR-Egger intercept analysis were applied for sensitivity analysis. IVW and cML-MA were considered the primary analytical tools, and the results of the other 4 MRs were used as complementary and validation. The results suggest that there is no significant causal relationship between leukocyte count and breast cancer risk (IVW OR = 0.98 [95% CI: 0.93–1.03], p-value = 0.35; CML-MA OR = 1.01 [95% CI: 0.98–1.05], p-value = 0.51). In addition, we analyzed whether there was a potential correlation between the five main types of categorized leukocyte counts and different breast cancer subtypes. We did not find significant evidence to support a significant correlation between leukocyte counts and breast cancer subtypes.

## Introduction

Breast cancer (BC) is a common female cancer, and due to its high mortality and morbidity, BC has become a significant burden on women’s health^1–3^. Notably, in 2018, the United States reported the diagnosis of approximately 268,670 new BC cases^4,5^. Moreover, BC exhibits pronounced heterogeneity, showcasing varying clinical presentations among individual patients, thus posing intricate challenges in its therapeutic approach. Drawing upon both molecular and histological evidence, BC may be systematically categorized into three primary subtypes: hormone receptor-positive BC (estrogen receptor (ER+) or progesterone receptor (PR+)), human epidermal receptor 2-positive BC (HER2+), and triple-negative breast cancer (TNBC)^6–8^.

The significance of the immune microenvironment in the initiation and advancement of BC has garnered growing recognition^9,10^. Tumor-associated immune cells have exhibited prospective prognostic utility across diverse malignancies, with elevated quantities of tumor-infiltrating lymphocytes frequently linked to more favorable clinical outcomes in BC^11^. However, research in the realm of the association between circulating immune cell subtypes and the clinical attributes or risk profiles of BC patients remains notably underexplored.

A patient’s leukocyte levels may provide clues to BC risk. A study by Kresovich et al.^12^ suggests that the percentage of certain leukocyte types in a woman’s blood may predict her risk of being diagnosed with breast cancer in the short and long term. The immune system plays an important role in the occurrence and development of BC, and it protects the health of the human body. Gene mutations are not uncommon in normal human beings, but not every harmful mutation will develop into a malignant tumor. This is because the immune system eliminates these mutant cells in real time and resolves the threat of cancer at the initial stage. Only when the immune system malfunctions or the mutated cells establish an immune escape mechanism can the mutated cells, which were originally very small, have the opportunity to continue to divide and proliferate, and eventually form tumors^13,14^. Therefore, detecting the immune function status of the body may be beneficial to assess the risk of malignant tumors such as BC^15,16^. White blood cells are important immune cells in the body, including neutrophils, eosinophils, basophils, lymphocytes and monocytes^17,18^. Different types of white blood cells participate in the body's immune defense response in different ways. A previous retrospective study has provided preliminary evidence of a conceivable correlation between peripheral leukocyte and neutrophil counts and the clinical attributes of BC patients^19^. Larsson et al.^20^ reported significant associations between peripheral blood mononuclear cell populations and prognosis in patients afflicted with metastatic BC. Furthermore, there is emerging evidence suggesting potential links between peripheral blood lymphocyte levels and clinical characteristics, as well as chemotherapy responsiveness, in BC patients^21^. The level of peripheral blood leukocyte count may be one of the most important indicators for evaluating the risk characteristics of BC patients, while the leukocyte categorical count may better reflect the dynamic characteristics of the microenvironment of BC patients.

**Figure 1:**
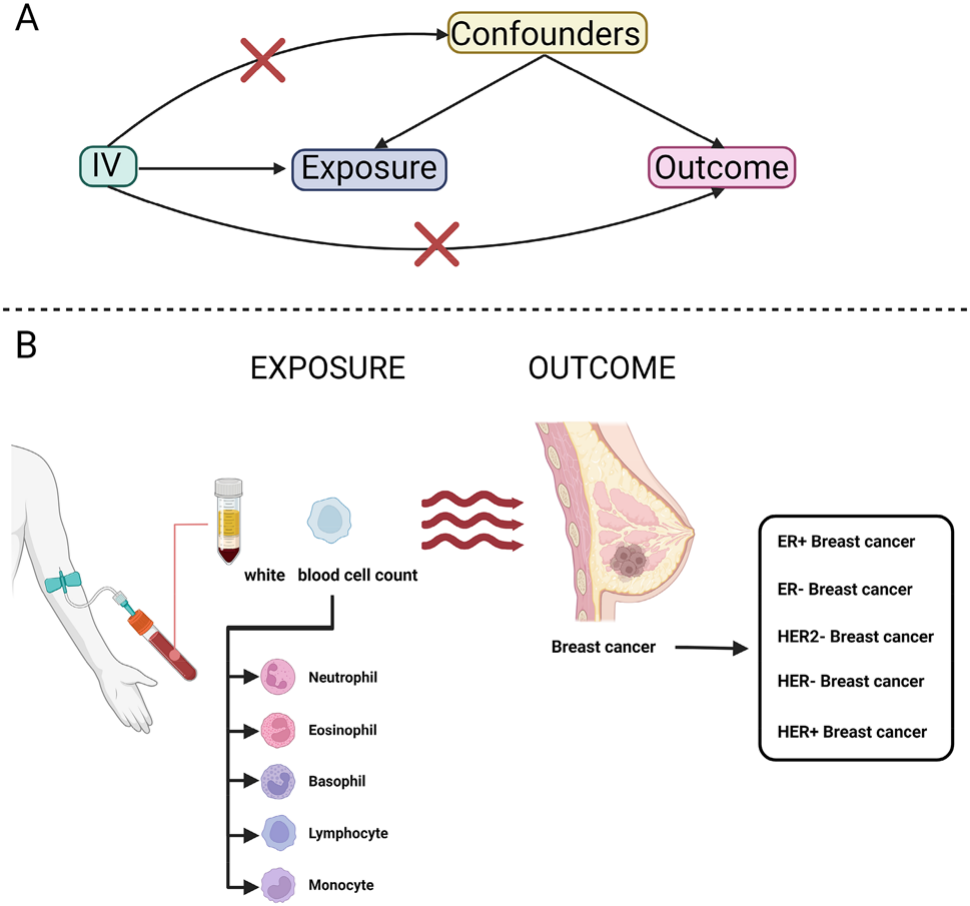
Research flow chart. (**A**) The selection of instrumental variables needed to fulfill 3 assumptions. (**B**) The exposure in the study was the white blood cells and their subtypes. The outcome in the study is breast cancer and its subtypes.

The majority of the research evidence regarding the association between peripheral blood leukocyte counts and BC risk is derived from observational studies. Nevertheless, these studies are susceptible to various limitations, including confounding factors, selection bias, and other biases that may compromise the validity of their findings. Mendelian randomization (MR) analysis employs genetic variants as instrumental variables (IVs) to enhance the causal inference in exposure-outcome relationships, particularly when pleiotropic effects are absent^22,23^. This approach mitigates the confounding issues inherent in observational epidemiology, ultimately yielding more dependable and rigorous results. MR is now widely used in the study of BC^24–26^. In this study, we performed MR analysis of two samples to investigate the potential association between peripheral blood leukocyte counts and breast cancer risk. In addition, we further investigated the potential relationship between categorized leukocyte cell counts (Neutrophils, eosinophils, basophils, lymphocytes and monocytes) and breast cancer subtypes (ER+ Breast cancer, ER− Breast cancer, HER− Breast cancer, HER+ Breast cancer, and HER2− Breast cancer). The flowchart of this study is shown in Fig. 1.

## Methods

### Study populations

“Exposure” in this study was defined as a categorical count of multiple leukocytes in peripheral blood, and the data were obtained from a GWAS study conducted by the Blood Cell Consortium (BCX)^27,28^. The study included 563,946 European ethnic subjects and provided GWAS data on five types of peripheral blood leukocyte counts: neutrophils, eosinophils, basophils, lymphocytes and monocytes (Supplementary Tables 1). In this study, “outcome” was selected as breast cancer and its subtypes, and data for Breast cancer, ER+ Breast cancer, and ER− Breast cancer were obtained from a published GWAS meta-analysis study conducted by the Breast Cancer Association Consortium (BCAC)^29^. Data for HER2− breast cancer, HER− breast cancer, and HER+ breast cancer were obtained from the FinnGen Consortium. The FinnGen study represents a research initiative that combines genetic data obtained from Finnish biobanks with health records derived from Finnish health registries^30^. All breast cancer cases had a diagnosis established according to standard procedures and were supported by a pathological basis. The exposure and outcome data were downloaded from the “GWAS summary statistics” database at https://gwas.mrcieu.ac.uk/^31,32^. Given that the present study utilized publicly available summary data, there was no necessity for additional ethical approval or participant consent. The exposure and outcome specific information is shown in Table 1.

**Table 1:** Characteristics of peripheral blood leukocytes and breast cancer data.

Type	GWAS ID	Trait	Source Institution	Race	Sample size	Year
Exposure	ieu-b-29	Basophil cell count	BCX	European	563,946	2020
	ieu-b-30	Leukocyte count	BCX	European	563,946	ieu-b-30
	ieu-b-31	Monocyte cell count	BCX	European	563,946	2020
	ieu-b-32	Lymphocyte cell count	BCX	European	563,946	2020
	ieu-b-33	Eosinophil cell count	BCX	European	563,946	2020
	ieu-b-34	Neutrophil cell count	BCX	European	563,946	2020
Outcome	ieu-a-1126	Breast cancer	BCAC	European	228,951	2017
	ieu-a-1127	ER+ Breast cancer	BCAC	European	175,475	2017
	ieu-a-1128	ER− Breast cancer	BCAC	European	127,442	2017
	finn-b-C3_BREAST_HER2NEG	HER2− Breast cancer	FinnGen	European	–	2021
	finn-b-C3_BREAST_HERNEG	HER− Breast cancer	FinnGen	European	–	2021
	finn-b-C3_BREAST_HERPLUS	HER+ Breast cancer	FinnGen	European	–	2021

BCX, blood cell consortium; BCAC, Breast Cancer Association Consortium.

### Selection of instrumental variables

Based on the relevance, independence and exclusivity assumptions of MR, single nucleotide polymorphism loci (SNPs, Single nucleotide polymorphisms) that met the requirements were screened from the above exposure GWAS data and used as instrumental variables (IV) in this study (Fig. 1A)^33,34^. Specifically, all SNPs had to meet the following criteria: (1) Exposure correlation P-value less than 5 × 10^–8^, along with an F-statistic greater than 10, thus satisfying the correlation assumption of MR. (2) SNPs with significant linkage disequilibrium with the measured SNPs (r2 = 0.001) were removed in the range of 10,000 kb, thus satisfying the independence assumption of MR. (3) SNPs with significant associations with outcomes or confounders were manually removed via the PhenoScanner website, thus satisfying the exclusivity hypothesis. These eligible instrumental variables were then extracted from the outcome GWAS with the necessary coordination so that the effect of a single instrumental variable on exposure corresponded to the same allele as the effect on outcome. The data of the IVs are shown in Supplementary data S1–S6.

### Statistical analysis

In this study, the Inverse-Variance Weighted (IVW), Weighted Median, MR-Egger, Maximum Likelihood (ML), MR-PRESSO and Constrained Maximum Likelihood and Model Averaging (cML-MA) methods of random effects models were used for MR analysis^35–38^. IVW is one of the most commonly used MR analysis methods^39,40^. The IVW method has the strongest causal inference and provides the most reliable findings when the instrumental variables are not confounded by horizontal pleiotropy. The weighted median and MR-Egger methods, on the other hand, serve as complements to the IVW method^41,42^. Because these two methods can be used under a wider range of conditions and provide more conservative results. Of these, the weighted median method allows no more than 50% of the instrumental variables to be confounded by horizontal pleiotropy, whereas the MR-Egger method allows all instrumental variables to be pleiotropic, but this pleiotropy cannot interfere with the correlation of instrumental variables with exposure^43^. In contrast to the IVW method, the ML method offers the advantage of a reduced standard error, and its findings remain unbiased in the absence of heterogeneity or horizontal polymorphism. We also used the MR-PRESSO method to identify and remove any outlier variants^44^. This method regresses the genetic variance results on the genetic variance exposure and uses residual squares to identify outliers. The cML-MA method, a MR technique that incorporates ML and model averaging, is applied to address both correlated and uncorrelated pleiotropic effects. Importantly, it does not rely on the InSIDE (Instrument Strength Independent of Direct Effect) assumption, setting it apart from other MR approaches. cML-MA has better type I error control. Xue et al.^45^ demonstrated that, in certain scenarios, the cML-MA method might offer advantages over the IVW method. As such, in this research, the primary analytical tools employed were the cML-MA and IVW methods, which were complemented by results obtained from four additional methods. MR-Egger and MR-PRESSO methods were employed to identify horizontal pleiotropy. In the MR-PRESSO analysis, the number of distributions was specified as 1000. Furthermore, the detection of heterogeneity was carried out using the IVW method and MR-Egger regression, with the quantification of heterogeneity achieved through the Cochran Q statistic. A P < 0.05 was considered statistically significant, while Bonferroni-corrected adjusted p-values (BP < 0.05/N, N = number of comparisons) in analyses involving multiple comparisons were considered statistically significant in order to minimize the potential risk of type I error and to improve the overall validity and interpretability of the results. The selection of all instrumental variables and the MR analysis process were done by applying the “TwoSampleMR” and “MRcML” R package (version 4.3.1)^45–48^.

### Ethics approval and consent to participate

Animal and human experiments were not conducted in this study.

## Results

### Analyzing the correlation between leukocyte counts and breast cancer risk

Previous studies have suggested that leukocyte counts in breast cancer patients may play a key role in breast cancer risk^12^. We further explored this by the method of MR analysis. IVW and cML-MA were considered the primary analytical tools, and the results of the other 4 MRs were used as complementary and validation. When neither IVW nor cML-MA was statistically significant, we considered that there was no causal relationship between exposure and outcome.

**Figure 2:**
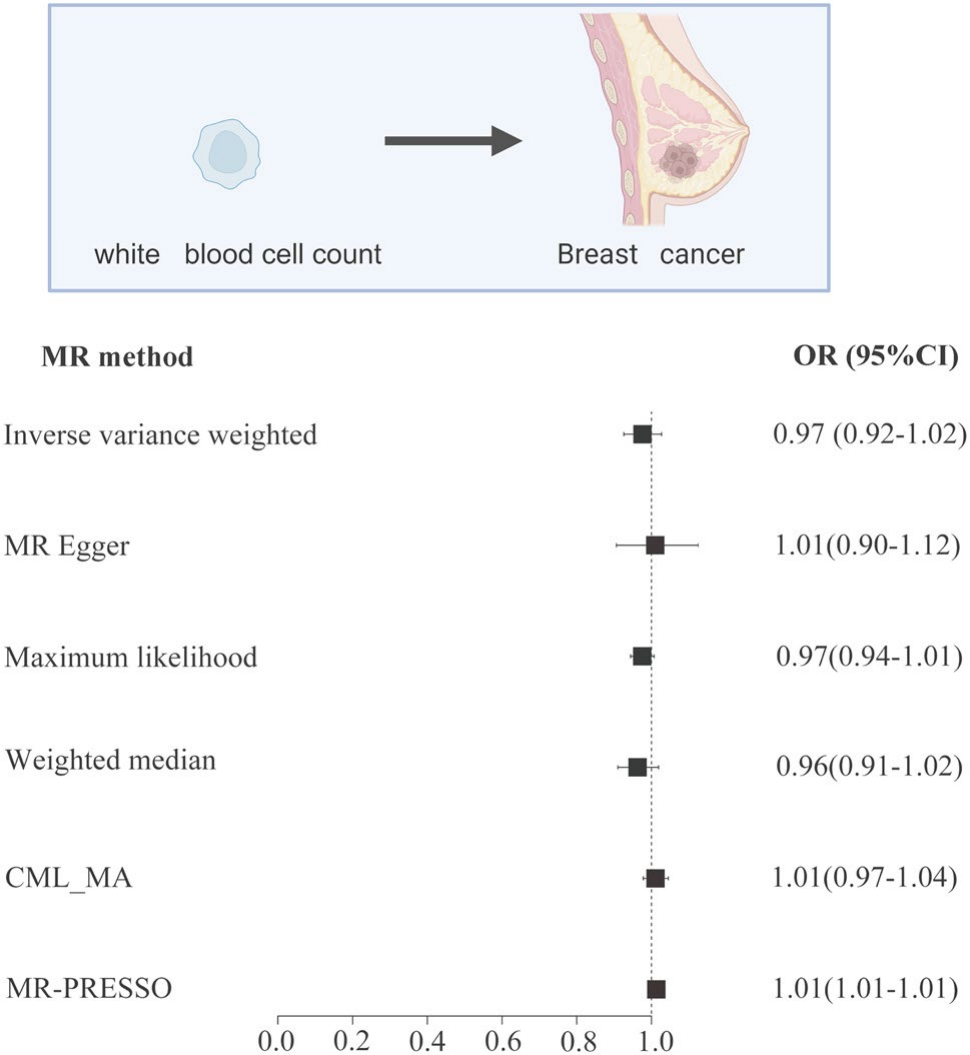
MR analysis between white blood cell count and breast cancer risk.

The results suggest that there is no significant causal relationship between leukocyte count and breast cancer risk (Fig. 2; Supplementary data S7; IVW OR = 0.98 [95% CI: 0.93–1.03], p-value = 0.35; CML-MA OR = 1.01 [95% CI: 0.98–1.05], p-value = 0.51). Cochran's Q-test showed that there was no heterogeneity among the IVs. The results of the MR-Egger regression intercept and the MR-PRESSO test showed that there was no horizontal pleiotropy among the IVs (P > 0.05).

**Figure 3:**
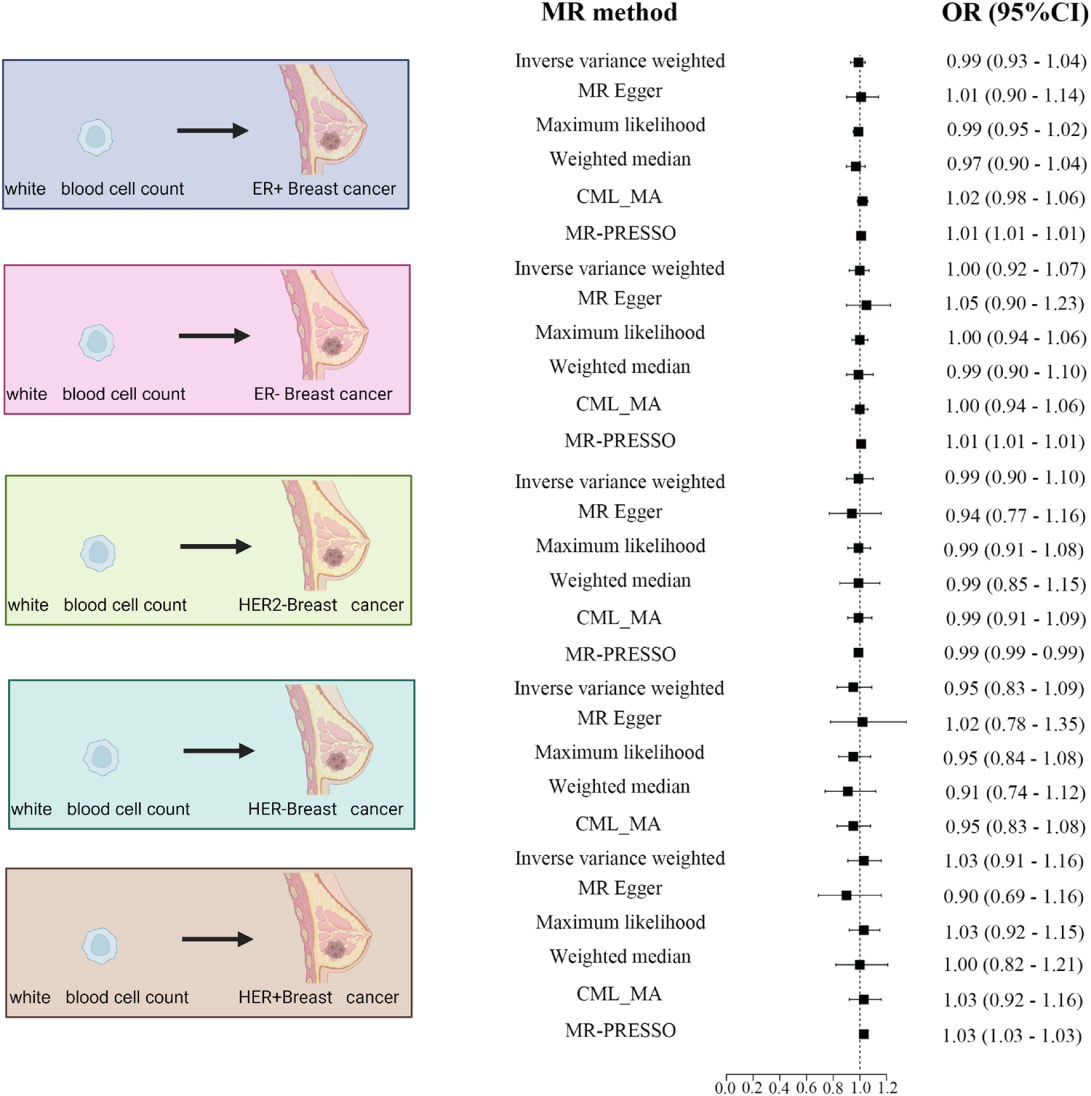
MR analysis of the association between white blood cell count and risk of breast cancer subtypes. The five common subtypes of breast cancer are shown from top to bottom. We used six different MR analyses, which will be labeled with one star when the P value is < 0.05 and two stars when the P value satisfies P = BP < 0.05/N. CI, confidence interval; OR, odds ratio.

In addition, we analyzed whether there was a potential correlation between leukocyte counts and different breast cancer subtypes (ER+ Breast cancer, ER− Breast cancer, HER2− breast cancer, HER- breast cancer, and HER+ breast cancer). We did not find significant evidence to support a significant correlation between leukocyte counts and breast cancer subtypes (Fig. 3; Supplementary Table S8; All MR methods had p-value > 0.05). Therefore, we further collected different types of leukocytes in the hope of finding evidence of a correlation with breast cancer risk in categorized leukocyte counts.

We used six different MR analyses, which will be labeled with one star when the P value is < 0.05 and two stars when the P value satisfies P = BP < 0.05/N. CI, confidence interval; OR, odds ratio.

### MR analysis of categorized white blood cell counts and breast cancer and its subtypes

**Figure 4:**
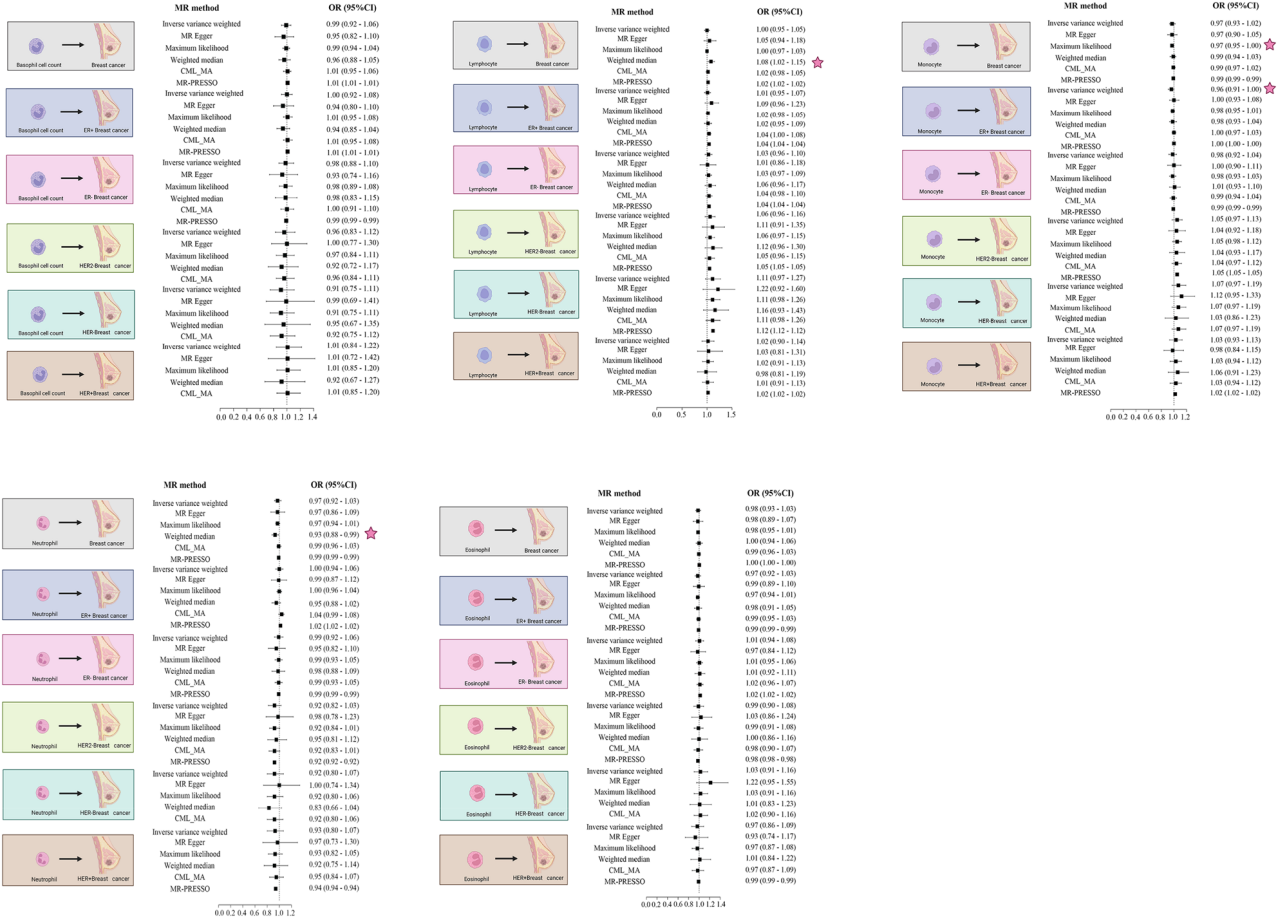
MR analysis of peripheral blood categorical leukocyte count and risk of breast cancer and its subtypes. Five different leukocyte types were analyzed by MR with breast cancer and its subtypes, respectively. We used six different MR analyses, which will be labeled with one star when the P value is < 0.05 and two stars when the P value satisfies P = BP < 0.05/N. CI, confidence interval; OR, odds ratio.

We further investigated the causal relationship between the five major leukocyte types and breast cancer and its subtypes (Fig. 4; Supplementary Table S9−S13). The results showed that no significant causal relationship was found between basophil and eosinophil counts and the risk of breast cancer and its subtypes. In all analyses, horizontal pleiotropy was absent (P > 0.05).

In addition, it is worth noting that the results of the weighted median method suggest a potential correlation between lymphocyte counts, neutrophil counts and breast cancer risk. However, the results of both IVW and CML-MA were not significant. Therefore, we do not consider this result to be significant.

Another important finding was that the results of IVW indicated a statistically significant correlation between elevated peripheral blood monocyte counts and reduced risk of ER+ breast cancer at the genetic level (OR = 0.96, 95% CI = 0.91–1.00, P = 0.043). However, considering multiple comparisons, we extended the P value to BP. the IVW result did not fulfill the requirement of BP, and therefore, this result was also not significant.

In conclusion, the results of MR analysis indicated that there was no significant correlation between the counts of peripheral blood leukocytes and their subtypes and the risk of breast cancer and its subtypes.

## Discussion

Breast cancer is a malignant tumor that poses a serious health risk to women^49^. Immune mechanisms play an obvious role in the occurrence and development of breast cancer. Therefore, in-depth studies targeting this area are beneficial to further reveal the pathogenesis of breast cancer and to improve its prevention and treatment measures. Peripheral blood leukocyte count is a routine clinical test, which is mainly used for the assessment of patients’ general condition and the diagnosis of acute bacterial infections, with the characteristics of being easy to perform and low cost^50,51^. As important immune cells in the organism, the levels of various leukocytes in peripheral blood can also reflect changes in immune function. Wei et al.^52^ recruited 140 Chinese women, 75 with breast cancer and 65 healthy controls. Their results showed that breast cancer patients had higher white blood cell counts, neutrophil counts and monocyte counts. Okutural et al.^53^ showed that neutrophil levels are associated with risk of breast cancer. A meta-analysis evaluated the association between neutrophil-to-lymphocyte ratio as a biomarker and breast cancer prognosis using leukocyte subtypes^54^. Therefore, the present study investigates the correlation between peripheral blood leukocyte sorting count levels and the risk of breast cancer development, giving researchers the opportunity to deepen their understanding of the immune mechanism of breast cancer development and clinicians the opportunity to find a simple method to assess the risk of breast cancer.

In this study we performed a large-scale MR analysis using six different MR analysis methods. We first investigated the potential relationship between leukocyte counts and the risk of breast cancer and its subtypes. The results of the MR analyses did not support a significant causal relationship between them. Then, we further investigated the relationship between leukocyte subtypes and breast cancer risk. The IVW results showed that there was a statistically significant correlation between an elevated peripheral blood monocyte count and a reduced risk of ER+ breast cancer (OR = 0.96, 95% CI = 0.91–1.00, P = 0.043). However, considering multiple comparisons, we extended the P-value to BP. The IVW results did not fulfill the requirement of BP, and therefore this result was also not significant. The results of our MR study showed no significant causal relationship between leukocyte count and breast cancer risk.

The present study is a two-sample MR study, which has the greatest advantage of avoiding causal reversal and minimizing the effect of confounding factors by using instrumental variables in place of phenotype for causal inference. The subjects of the breast cancer GWAS data in this study were all female, whereas the leukocyte GWAS data in this study were from a population of both sexes. This may affect the reliability of the study results to some extent. Current data do not support making gender distinctions. The results of previous studies have shown that a patient's leukocyte level can be affected by a variety of factors, such as time of diagnosis, body weight, hormone levels, and menopausal status^12,55^. In a recent study, Farrell et al.^56^ collected clinical characteristics of 37,052 men and 15,004 women, and their results indicated that the white blood cell count levels were approximately 6.0 ± 1.4 (10^9^/L) in men and 5.9 ± 1.5 (10^9^/L) in women. Gender may not be the main influencing factor of white blood cell count. The patient's body mass index and age may be more important influencing factors.

Overall, the results of the MR study showed no significant correlation between white blood cell counts and breast cancer risk. The results of this paper need to be further validated in clinical trials and in larger patient cohorts.

### Supplementary Information

Supplementary Legends.

Supplementary Table 1.

Supplementary Information 3.

Supplementary Information 4.

Supplementary Information 5.

Supplementary Information 6.

Supplementary Information 7.

Supplementary Information 8.

Supplementary Information 9.

Supplementary Information 10.

Supplementary Information 11.

Supplementary Information 12.

Supplementary Information 13.

Supplementary Information 14.

Supplementary Information 15.

## Data Availability

The original contributions presented in the study are included in the article/Supplementary Material, further inquiries can be directed to the corresponding author.
