# Supplementary material for: A Mendelian randomization-based approach to explore the relationship between leukocyte counts and breast cancer risk in European ethnic groups
Source: Sci Rep. 2023 Oct 9;13:16979. doi: 10.1038/s41598-023-44397-9 (PMC10562486; doi:10.1038/s41598-023-44397-9)
Supplement: Supplementary file 1 — Supplementary Legends. [file 41598_2023_44397_MOESM1_ESM.docx]

**Supplementary data:**

Supplemental Table 1. Summary of measurement methods for White blood cell count phenotypes.

Supplementary data S1. IV-Basophil count.

Supplementary data S2. IV-Eosinophil count.

Supplementary data S3. IV-Lymphocyte count.

Supplementary data S4. IV-Monocyte count.

Supplementary data S5. IV-Neutrophil count.

Supplementary data S6. IV-leukocyte count.

Supplementary data S7. MR analysis results of leukocyte count and breast cancer risk.

Supplementary data S8. MR analysis results of leukocyte count and different breast cancer subtypes.

Supplementary data S9. MR analysis results of basophil cell count and different breast cancer subtypes.

Supplementary data S10. MR analysis results of monocyte cell count and different breast cancer subtypes.

Supplementary data S11. MR analysis results of lymphocyte cell count and different breast cancer subtypes.

Supplementary data S12. MR analysis results of eosinophil cell count and different breast cancer subtypes.

Supplementary data S13. MR analysis results of neutrophil cell count and different breast cancer subtypes.
