# Supplementary material for: A Mendelian randomization-based approach to explore the relationship between leukocyte counts and breast cancer risk in European ethnic groups
Source: Sci Rep. 2023 Oct 9;13:16979. doi: 10.1038/s41598-023-44397-9 (PMC10562486; doi:10.1038/s41598-023-44397-9)
Supplement: Supplementary file 2 — Supplementary Table 1. [file 41598_2023_44397_MOESM2_ESM.docx]

**Supplemental Table 1. Summary of measurement methods for White blood cell count phenotypes.**

| Cell Type | Long Name | Unit | Description | Determination |
| --- | --- | --- | --- | --- |
| Compound white cell | White blood cell count | per nL | Aggregate count of white cells per unit volume of blood | Impedance |
|  | Monocyte percentage of white cells | % | Percentage of white cells that are monocytes | Flow cytometry gates |
|  | Neutrophil percentage of white cells | % | Percentage of white cells that are neutrophils | Flow cytometry gates |
|  | Eosinophil percentage of white cells | % | Percentage of white cells that are eosinophils | Flow cytometry gates |
|  | Basophil percentage of white cells | % | Percentage of white cells that are basophils | Flow cytometry gates |
|  | Lymphocyte percentage of white cells | % | Percentage of white cells that are lymphocytes | Flow cytometry gates |
